# Noniterative Generalized Camera Model for Near-Central Camera System

**DOI:** 10.3390/s23115294

**Published:** 2023-06-02

**Authors:** Taehyeon Choi, Seongwook Yoon, Jaehyun Kim, Sanghoon Sull

**Affiliations:** School of Electrical Engineering, Korea University, 145 Anam-ro, Seongbuk-gu, Seoul 02841, Republic of Korea; taehyeon@korea.ac.kr (T.C.); swyoon@mpeg.korea.ac.kr (S.Y.); jhkim@mpeg.korea.ac.kr (J.K.)

**Keywords:** camera calibration, generalized camera model, 3D measure, transparent shield, stereo camera calibration, fisheye camera model, catadioptric camera model, refraction of ray near-central camera model

## Abstract

This paper proposes a near-central camera model and its solution approach. ’Near-central’ refers to cases in which the rays do not converge to a point and do not have severely arbitrary directions (non-central cases). Conventional calibration methods are difficult to apply in such cases. Although the generalized camera model can be applied, dense observation points are required for accurate calibration. Moreover, this approach is computationally expensive in the iterative projection framework. We developed a noniterative ray correction method based on sparse observation points to address this problem. First, we established a smoothed three-dimensional (3D) residual framework using a backbone to avoid using the iterative framework. Second, we interpolated the residual by applying local inverse distance weighting on the nearest neighbor of a given point. Specifically, we prevented excessive computation and the deterioration in accuracy that may occur in inverse projection through the 3D smoothed residual vectors. Moreover, the 3D vectors can represent the ray directions more accurately than the 2D entities. Synthetic experiments show that the proposed method can achieve prompt and accurate calibration. The depth error is reduced by approximately 63% in the bumpy shield dataset, and the proposed approach is noted to be two digits faster than the iterative methods.

## 1. Introduction

Various camera models and corresponding calibration methods have been developed to identify the relation between a two-dimensional (2D) image pixel and three-dimensional (3D) world point. Generally, this relation is defined as a 3D-to-2D perspective projection. Foley et al. [1], Brown et al. [2], and Zhang et al. [3] proposed a pinhole camera model with radial and tangential distortions of real lens models, which is widely used in camera calibration. Scaramuzza et al. [4] and Kannala et al. [5] modeled a fisheye camera using polynomial terms with additional distortion terms. Usenko et al. [6] proposed a double-sphere model with a closed form of both projection and back-projection procedures to reduce the computational complexity in projections. Jin et al. [7] showed that camera calibration requires a large number of images, although a point-to-point can be used to realize calibration using dozens of images.

Tezaur et al. [8] analyzed the ray characteristic of a fisheye lens, in which the rays converge on one axis. The authors extended the fisheye camera model of Scaramuzza et al. [4] and Kannala et al. [5] by adding terms to compensate for the convergence assumption of the model.

Notably, the abovementioned methods can only be applied to simple camera systems. Other methods have been developed to consider more complex systems, such as transparent shields or cameras behind reflective mirrors. Treibitz et al. [9] explicitly modeled a flat transparent shield. Additionally, Yoon et al. [10] used an explicit model that can be applied from a flat to a curved shield. Pável et al. [11] used the radial basis functions to model uneven outer surfaces of the shield.

Geyer et al. [12] used unifying models for various configurations of a catadioptric camera, which is a camera model that contains various lenses and mirrors. When the camera and mirror are aligned, the rays converge at one point; otherwise, the rays do not converge. However, this model cannot handle the case in which misalignment occurs between the camera and lens. The model proposed by Xiang et al. [13] can address such misalignments.

Certain other models can implicitly model the distortions caused by a transparent shield or reflective mirrors. For example, the fisheye camera model of Scaramuzza et al. [4] can be used for catadioptric cameras, in which implicit modeling is realized using polynomial terms.

The generalized camera model [14] defines a raxel as a 1:1 mapping relationship between the image pixels and rays. This model can be categorized as central or non-central depending on the ray configuration. The central camera model assumes that the rays converge at one point. Ramalingam et al. [15] used multiple 2D calibration patterns to alleviate the requirement for dense observations. The authors compensated for the sparsity using interpolation. Rosebrock et al. [16] and Beck et al. [17] used splines to obtain better interpolation results. The models of Nishimura et al. [18], Uhlig et al. [19], and Schops et al. [20] do not assume ray convergence and are thus non-central camera models.

In extended uses of cameras, when the camera is behind a transparent shield or reflective object, the induced distortion is more complicated and thus more complex models are required.

Compared with 2D observations, 3D observations are preferable for simplifying the calibration process and yielding more accurate calibration results. Although 3D observation devices such as Lidar and Vicon sensors were used only for industrial purposes in the past, they are being widely used by general users at present owing to their decreased costs and enhanced performance.

Several methods have been developed using 3D observations. Miraldo et al. [21] improved the generalized camera model. Verbiest et al. [22] observed the object through a pinhole camera behind a windshield. Both methods are effective if the neighboring points are smooth. However, the accuracy may decrease or the computational complexity may increase in the following cases:

- Near-central scenarios;
- Cases in which the distance between the observation points and camera increases;
- Cases in which the number of observation points increases.

**Figure 1:**
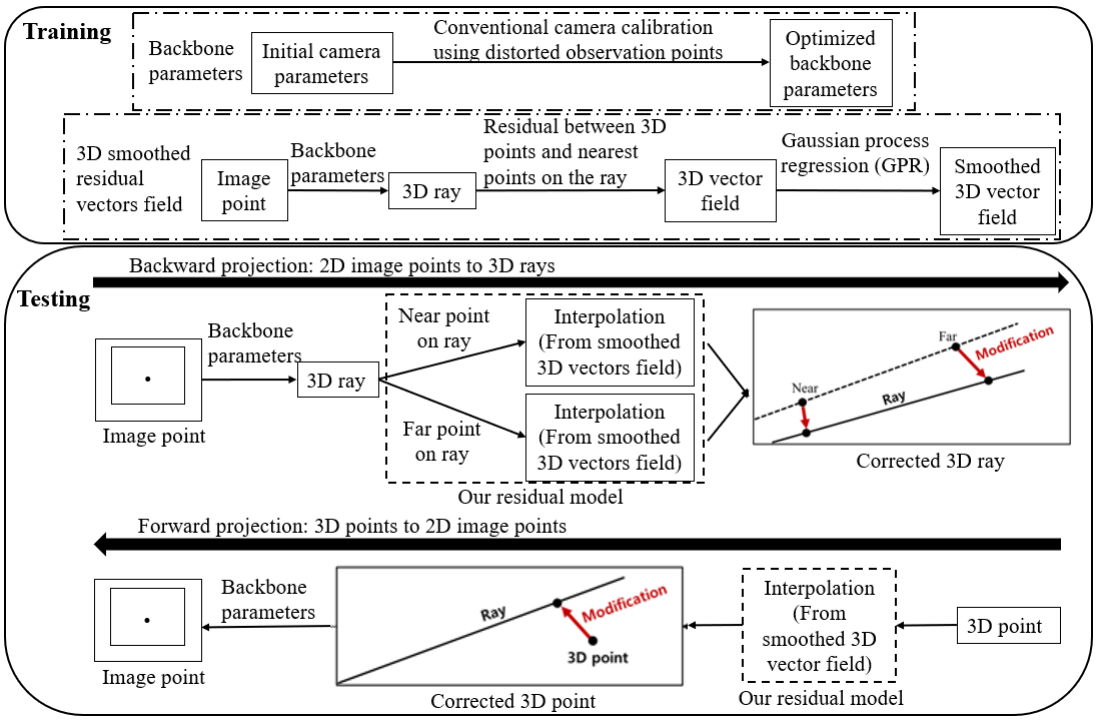
Process flow of the proposed camera model.

We propose a camera model that can be applied to the above mentioned cases. As shown in Figure 1, the backbone parameters and 3D residual vectors were calculated in the training process and used for the calibration in the testing process. The proposed camera model is divided into a backbone and residual model. The backbone can be any central camera model, and the residual model estimates the residual of the backbone model. The objectives of this study can be summarized as follows:

- The development of a near-central camera model;
- The realization of noniterative projection by the 3D residual model;
- The establishment of a simple calibration method suitable for sparse and noisy observations.

The proposed frameworks were verified by performing experiments in near-central scenarios: (1) when the camera is behind a transparent shield with noise, and (2) when the lenses or mirrors of fisheye or omnidirectional cameras are misaligned and noisy. Notably, we have strengths in two scenarios. First, when the shield’s exterior is subjected to external pressure or heat, the interior remains unchanged, but the outer surface is deformed by approximately 3–6 mm. Therefore, the shape appears as slightly bumpy. Second, for the same reasons as before, $1^{\circ }$ or 1 mm misalignment can occur in fisheye or omnidirectional camera systems.

**Figure 2:**
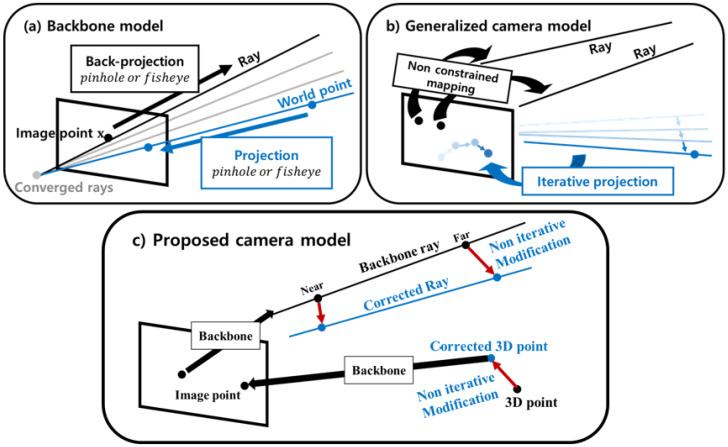
Differences in the existing and proposed camera models.

In the above mentioned cases, we can use the simple central camera model as the backbone. The use of this model facilitates the optimization and prompt computation of the forward and backward projection processes. Moreover, as shown in Figure 2c, we can perform noniterative calibration by simply calibrating the ray through the 3D residual framework.

The remaining paper is organized as follows. Section 2 describes the proposed approach. Section 3 present the experimental results, and Section 4 presents the concluding remarks.

## 2. Proposed Camera Model

We define the two sub-models and describe the calibration process. Moreover, we describe the application of the proposed camera model to the forward and backward projection processes.

### 2.1. Definition

The proposed camera model consists of two sub-models: the backbone and residual models. The backbone model may be one of the central camera models, such as the simple pinhole or fisheye camera model. The projection and backward projection of the backbone model can be expressed as follows:

(1) $$\begin{array}{cc} x & =b(w) \end{array}$$

(2) $$\begin{array}{cc} R & =\left[0,\tilde{w}\right],\tilde{w}\in b^{-1}\left(x\right), \end{array}$$

where b(·) is a forward projection of the backbone model, and $b^{-1}\left(\cdot \right)$ is the inverse process of b(·). x and w are image and world points, respectively. R is a ray represented by two 3D points that the ray passes through.

The forward and backward projections for generalized camera models can be represented by g(·) and $g^{-1}\left(\cdot \right)$, respectively:

(3) $$\begin{array}{cc} x & =g^{-1}\left(R\right) \end{array}$$

(4) $$\begin{array}{cc} R & =g(x). \end{array}$$

The generalized camera model involves the intricate process $g^{-1}\left(\cdot \right)$, which results in an iterative procedure, the complexity of which increases with a decrease in the smoothness of g(·). Because we used a pinhole or fisheye camera model as the backbone model, we can use the simple projection or backward projection processes of the backbone model.

The residual model compensates for the error. The backbone with a 2D residual model can be represented as follows:

(5) $$\begin{array}{cc} x & =b(w)+r(w) \end{array}$$

(6) $$\begin{array}{cc} R & =[{\tilde{w}}_{1},{\tilde{w}}_{2}], \end{array}$$

(7) $$\begin{array}{cc} \; & {\tilde{w}}_{1},{\tilde{w}}_{2}\in f^{-1}\left(x\right), \end{array}$$

where r(·) is the residual model of the backbone model. The projection result of the backbone model is modified in an image plane. $f^{-1}\left(\cdot \right)$ is the inverse process of Equation (5). This inverse process is iterative, requiring the model to find two world points projected onto the same image point. A representative example is the model proposed by Verbiest et al. [22]. This model uses the cubic spline, which involves four spline functions for a single point. Because the process must be repeatedly performed for each step in the forward projection, it is computationally expensive.

Unlike the 2D residual, we used the 3D residual, which can be defined as follows:

(8) $$\begin{array}{cc} x & =b(w+r(w)); \end{array}$$

(9) $$\begin{array}{cc} R & =[{\tilde{w}}_{1},{\tilde{w}}_{2}], \end{array}$$

(10) $$\begin{array}{cc} \; & {\tilde{w}}_{1},{\tilde{w}}_{2}\in f^{-1}\left(x\right). \end{array}$$

Similar to the 2D residual, the inverse process $f^{-1}\left(\cdot \right)$ involves an intricate procedure. Specifically, it involves the iterative process of finding two world points that are projected onto one image point. To make the model simple and noniterative, we introduced the following backward process:

(11) $$\begin{array}{cc} R & =[{\tilde{w}}_{1}+r^{\prime }\left({\tilde{w}}_{1}\right),{\tilde{w}}_{2}+r^{\prime }\left({\tilde{w}}_{2}\right)], \end{array}$$

(12) $$\begin{array}{cc} \; & {\tilde{w}}_{1},{\tilde{w}}_{2}\in b^{-1}\left(x\right), \end{array}$$

where $r^{\prime }$ is the residual model for the backward projection. We substituted the computationally expensive iterative process of the backward projection of $f^{-1}\left(\cdot \right)$ to the two simple modifications of the backward residual model $r^{\prime }$. We used the residual model to modify the ray from a backbone model. The ray was modified by modifying two points on the ray and obtaining the ray that passes through the two modified points.

The residual model of backward projection $r^{\prime }$ is simply an inverse residual of the forward projection, represented as follows:

(13) $$\begin{array}{cc} r(w) & =interp(w;F) \end{array}$$

(14) $$\begin{array}{cc} r^{\prime }\left(w\right) & =interp(w;F^{-1}), \end{array}$$

where F is a sparse 3D error vector field of the backbone model, which is calculated over calibration data. Because the calibration data are not dense, interpolation must be performed to estimate the residual. The determination of F is described in Section 2.3.

In the following sections, we describe the calibration process of the backbone and residual models. Although the proposed camera model can jointly optimize both the backbone and residual models, an adequate performance can be achieved by two simple calibration procedures, as discussed herein.

### 2.2. Calibration of the Backbone Model

Verbiest et al. [22] proposed a backbone model that corresponds to a real camera model behind a windshield. In contrast, our backbone model is a simple central camera model with parameters optimized on training data. For example, when a transparent shield is present in front of the camera, we use the pinhole camera model as the backbone and optimize the parameters assuming the absence of the transparent shield. We used a simple central camera model to ensure a smooth residual that can be easily estimated even in situations involving sparse calibration data. Moreover, we optimized the parameters to fit into the calibration data to maximize the standalone performance of the backbone model.

Before optimizing the parameters of the backbone model, we assumed that the initial estimates of the parameters are available. This assumption is reasonable because the manufacturers’ specifications for a camera can be easily obtained. From the initial parameters of the pinhole or fisheye camera, we updated the parameters using gradient descent to minimize the reprojection error.

### 2.3. Calibration of the Residual Model

The calibration of the residual model is reduced to finding the following sparse 3D error vector field:

(15) $$\begin{array}{cc} F & =\left\{\left(w,p_{w}\left(b^{-1}\left(x\right)\right)-w\right)|\left(x,w\right)\in X\right\} \end{array}$$

where F is a sparse vector field and $p_{w}\left(b^{-1}\left(x\right)\right)$ is the nearest point on the ray of the backbone model to the 3D point w. In backward projection, the sparse vector field $F^{-1}$ is a reversed version of F, with the start point being $p_{w}\left(b^{-1}\left(x\right)\right)$ and the vector being w − $p_{w}\left(b^{-1}\left(x\right)\right)$.

### 2.4. Methodology

#### 2.4.1. Smoothing of Sparse 3D Vectors Field

**Figure 3:**
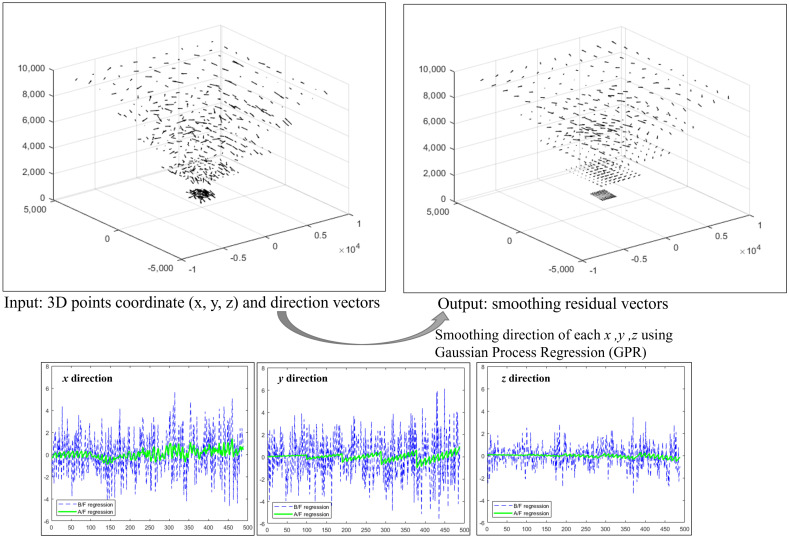
Generating the smoothed 3D vectors field.

Because the observation data have noise, the sparse vector field should be smoothed to ensure robustness against noise. As shown in Figure 3, we used three independent Gaussian regression processes for each direction of the residual vector in 3D.

#### 2.4.2. Interpolation

We used simple interpolation to obtain a dense vector field from the smoothed sparse vector field, considering fast forward and backward processes. To capture the local error structure in the training data, inverse distance weighting on nearest neighbors was applied for interpolation interp(w;F):

(16) $$r\left(w\right)=\frac{1}{Z_{w}}\sum_{w_{i}\in N_{w}}\frac{1}{||w-w_{i}{\left|\right|}_{2}}\left(p_{w_{i}}\left(b^{-1}\left(x\right)\right)-w_{i}\right),$$

where $Z_{w}$ and $N_{w}$ are the normalizing constant and number of neighbors for w, respectively. $r^{\prime }\left(w\right)$ was obtained by interpolating $F^{-1}$.

In addition, we calculated the magnitude of the vectors of the 3D residual to enable adaptive interpolation. If the magnitude of the residual vectors is large (small), a small (large) number of vectors are used for the interpolation.

#### 2.4.3. Straightness of the Back-Projected Ray

**Figure 4:**
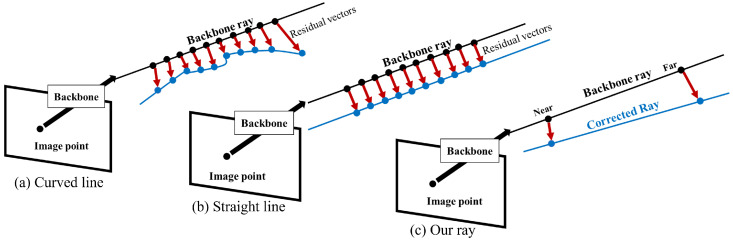
Our ray calibration method.

Using the 3D error vector field, the most intuitive strategy to modify the backbone ray is to modify every point on the ray. However, the modified ray will not be a straight line, as shown in Figure 4a; this is because the magnitude of the residual vectors has various values. This modification is invalid because the world points corresponding to one pixel should lie in a straight line. However, as shown in Figure 4b, we can solve this problem by constraining the construction of the residual vectors to have the same size.

However, we did not use this constraint because we tried to capture the local error of the backbone model. We introduced a relaxed constraint to capture the local error effectively and consider the noise in the calibration data.

Thus, using the 3D vector field, we prepared a backward model depending on the depth. This backward model outputs not a ray but a curve in 3D. The residual vector compensates for errors at a specific 3D point for each depth.

However, many existing computer-vision algorithms require the camera model to yield a ray. Therefore, to ensure the usability of the camera model, we designed a camera model that outputs a ray from the backward projection.

As shown in Figure 4c, we prepared a ray from two modified points, each sampled from the near and far points of the backbone ray, where the range is within the area in which the calibration data exist. For more clarity, the estimated ray in the proposed camera model refers to the corrected ray. To evaluate the estimated ray, we measured its distance from the 3D ground truth point. This distance was calculated by measuring the distance between two points: one is the 3D GT point, and the other one is the closest point on the ray to the GT 3D.

## 3. Experiments

The proposed frameworks were verified by performing experiments involving near-central cases: a pinhole camera behind a transparent shield, a fisheye camera, and a catadioptric camera with a misaligned lens.

**Figure 5:**
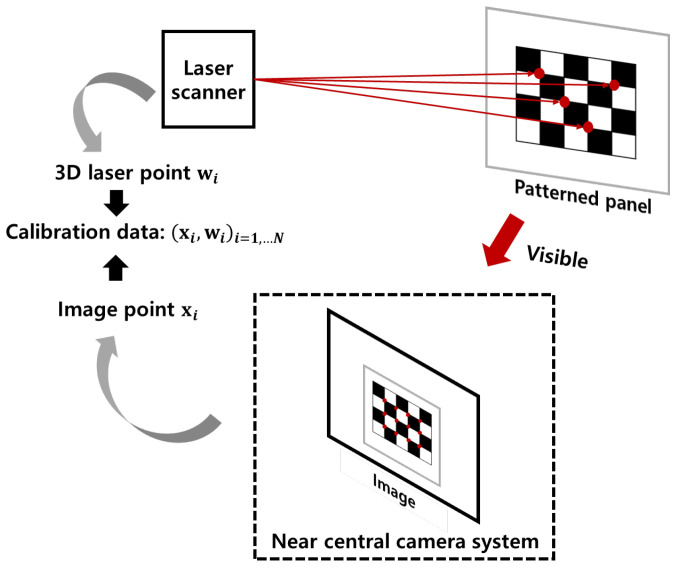
The acquisition process of calibration data. The laser scanner shoots the laser to the patterned panel, and then the position of 3D points can be obtained by the laser scanner. Two-dimensional image points corresponding to the three-dimensional points in the panel can be obtained by the image of the near-central camera system. When the panel is positioned far away from the camera, using a non-patterned panel may cause the laser point to become invisible to the near central camera system. To overcome this issue, a patterned panel can be used instead. By directing the laser to the corner point of the patterned panel, it becomes possible to obtain the 2D image point corresponding to the 3D laser point.

To ensure the accurate acquisition of 3D points, we assumed that the data could be obtained from a laser scanner. We simulated the data acquisition process using the laser scanner in Verbiest et al. [22], shown in Figure 5. The laser scanner is outside of the near-central camera, e.g., outside of the transparent shield, so that it is not affected by the shield. Then, the laser scanner shoots the laser toward the patterned panels, and the position of the 3D points in the panel can be obtained by the laser scanner. The 3D laser point in the panel can be visible in the near-central camera, so the position of the 2D image point of the corresponding 3D laser point can be obtained. The pairs of 2D image points and 3D laser points are the calibration (training) data. We simulated this process to obtain the data.

Since the proposed camera model compensates for the error of the backbone model using interpolation of a sparse 3D residual vector field from training 3D points, the proposed camera model operates on the range of training 3D points. For this reason, we evaluated the performance of the proposed camera model at the 3D points that have the same range as training 3D points, but are more dense than training data.

We evaluated the performance of the calibration methods using the reprojection error, the distance between the estimated ray and ground truth (GT) point, and the depth. We calculated the absolute average error (MAE) of the three metrics, and each metric is measured as follows:

- Reprojection error is a metric used to assess the accuracy of the forward projection by measuring the Euclidean distance between the reprojected estimation of a model and its corresponding true projection.
- The Euclidean distance between the estimated ray and the ground truth (GT) point serves as a metric for evaluating the accuracy of the backward projection. This involves locating the nearest point on the estimated ray when provided with the GT point and calculating the distance between these two points.
- For the depth error evaluation, a straight line was drawn connecting the ray estimated from the left camera to the ray estimated from the right camera. The midpoint of this straight line represents the estimated depth. The depth error was determined by calculating the distance between the estimated depth and the GT point.
- Lastly, relative error rates were calculated to allow for comparison with other methods. The formula for calculating the relative error ϵ is as follows:
  (17) $$\begin{array}{c} \epsilon =\frac{\alpha -\beta }{\alpha }\times 100, \end{array}$$
  where α is each existing method and β is the proposed method.

To make the experimental environment similar to a real environment, the evaluations were performed at different noise levels. To consider random noise, ten experiments were performed at the same noise level, and the MAE was measured as the final value to ensure a fair evaluation. Moreover, we added random noise to the image and 3D laser points in the training data.

### 3.1. Camera behind a Transparent Shield

**Figure 6:**
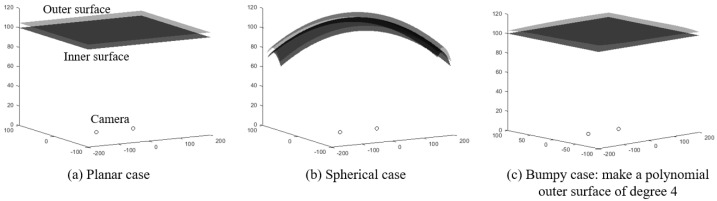
Three cases involving a transparent shield.

Three shield shapes were considered in this study: a plane, a sphere, and a hybrid shape with an inner plane surface and a slightly bumpy outer surface (approximately 3–6 mm). These shields have a uniform thickness of 5 mm and represent real-world scenarios, such as car windshields or camera shields for submarines or spaceships. Figure 6c illustrates the third shape.

The camera used in the experiment has a focal length *f* of 1000 (in pixels), with the principal point located at $c_{x}$ = 960, $c_{y}$ = 540 (in pixels). The camera does not exhibit any radial or tangential distortions. The image has a width of 1920 (in pixels) and a height of 1080 (in pixels).

Depth estimation was performed using a stereo camera setup for all three shield types. The baseline distance between the left and right cameras is 120 mm, and both cameras share the same intrinsic parameters.

#### 3.1.1. Calibration Data

Three-dimensional points were extracted from 1000 mm to 10,000 mm, with 1000 mm and 2000 mm intervals for the test and training datasets. Consequently, the training data comprised approximately 10% of the test data. The noise level with a random normal distribution of μ=0 and from σ=0 to σ=1.0 was added to the image points, and noise with a random normal distribution of μ=0 and from σ=0 to σ=10 was added to the 3D laser points.

#### 3.1.2. Results for the Transparent Shield Case

**Figure 7:**
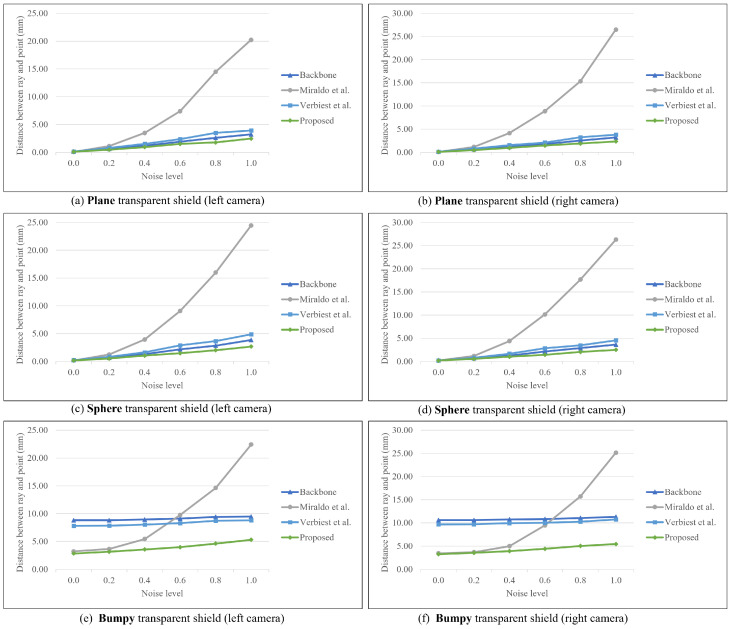
Results of distance for planar, spherical, and bumpy shields [21,22].

The experimental results for shields with different shapes are presented in Table 1 and Figure 7. The errors for planar and spherical shields are lower compared to the bumpy shield, and the error increases with the noise level. We add explanations about the calibration data at the beginning of the experiment section. The bumpy shield exhibits minimal sensitivity to noise, possibly due to the optimization of backbone parameters considering the shield’s characteristics. Determining the effect of noise is challenging due to the substantial distortion caused by refraction. The proposed method outperforms others as the noise level increases since most existing approaches focus on minimizing the reprojection error in 2D space, whereas the proposed method enables direct calibration in 3D space.

The important hyperparameters of the two methods, control point and patch size, were set as 10 and 4 by 4, respectively, because they correspond to the lowest error. The method proposed by Miraldo et al. [21] has an excellent performance in the case of low-level noise. However, at high noise levels, their approach has a considerable error compared with those of other methods. Notably, this approach parameterizes the ray with the Pl$\ddot{u}$cker coordinate, composed of two orthogonal vectors, moment and direction. The moments and directions are optimized to minimize the objective of the distance between the 3D point and corrected ray.

However, the objective is only valid when the estimated vectors (moment and direction) are orthogonal. After optimization, we checked the orthogonality of the two vectors, as presented in Table 2. The results show that the average angle difference between two vectors is more than $90^{\circ }$ at high noise levels. Thus, significant errors are induced when noise is added.

**Figure 8:**
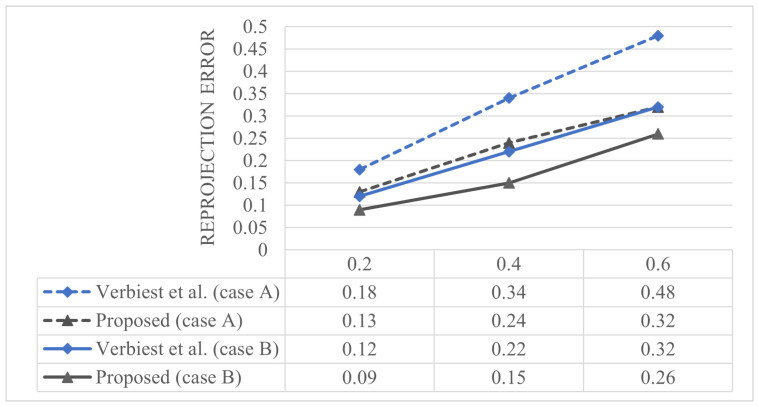
Comparison of the reprojection error by increasing the number of training points [22].

Verbiest et al. [22] modeled the spherical shield and used more training points than test points. In contrast, our datasets have denser test points than training points; therefore, we increased the number of training points to ensure a fair comparison, as shown in Figure 8. Case A corresponds to the same environment as that presented in Table 1, with 486 training points and 3913 test points. Case B has 1839 training points and 1535 test points. We can show that the reprojection error of case B is lower than that in case A; however, this observation is meaningless because another method was also improved.

**Figure 9:**
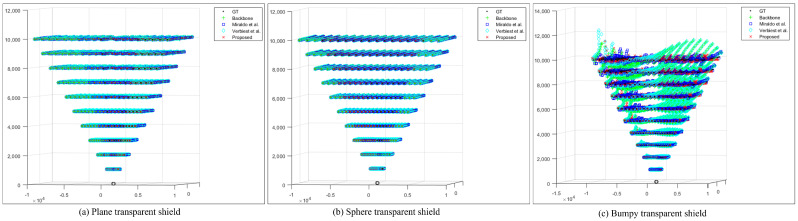
Results of the depth for planar, spherical, and bumpy shields [21,22].

Figure 9 shows the depth results. To examine the depth results for different shapes, the results of noise level zero in Table 1 are visualized. In the case of planar and spherical shields, the differences across the methods are insignificant. However, in the case of the bumpy shield, the results of the proposed method are the closest to GT points. The planar and spherical shields exhibit minor errors; however, in the case of the bumpy shield, the gap between the rays of the left and right cameras is widened, and thus errors are present even after calibration.

### 3.2. Fisheye Camera with Misaligned Lens

Second, we simulate dthe fisheye camera, which has the following internal parameters. The polynomial coefficients for the projection function are described by the Taylor model proposed by Scaramuzza [4] and specified as an [348.35, 0, 0, 0] vector. The center of distortion in the pixels is specified as a [500, 500] vector, and the image size is 1000×1000 pixels.

#### 3.2.1. Calibration Data

**Figure 10:**
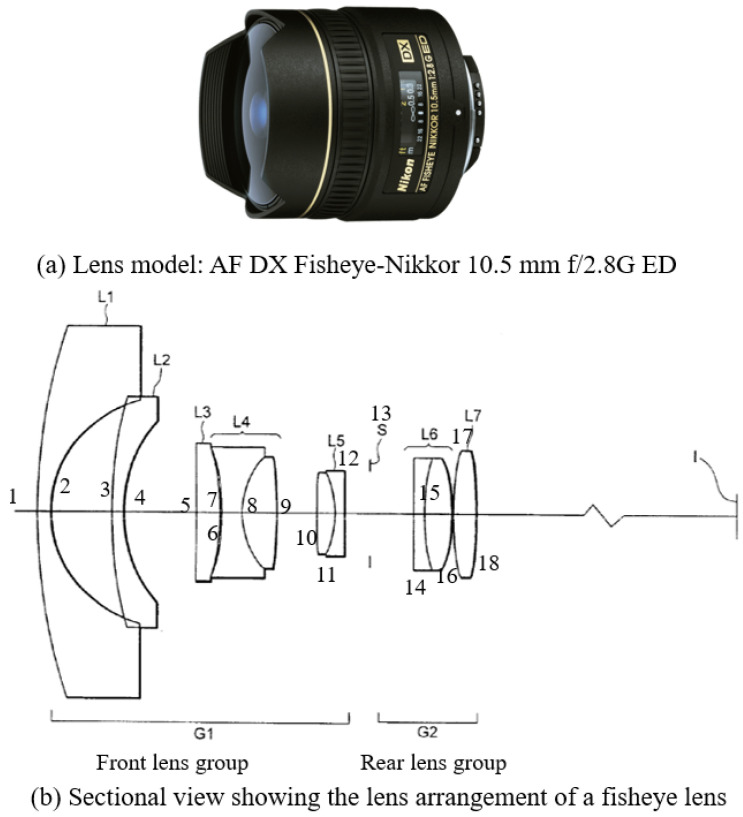
Reference fisheye lens model [24].

To simulate the fisheye lens, we used the Zemax OpticStudio [23] software, which is widely used in the optics field. We simulated the lens using the specifications presented in Figure 10a, referenced from Keiko Mizuguchi’s patent Fisheye lens [24].

In the misaligned case #1, we tilted the L2 lens $1^{\circ }$ for the left camera and L2 lens $-1^{\circ }$ for the right camera as shown in Figure 10b. In the case #2, the L1 lens was shifted by 1 mm and the L2 lens was tilted by $1^{\circ }$ for the left camera. In the right camera, the L1 lens was tilted by $1^{\circ }$, and the L2 lens was shifted by 1 mm. The distance from the camera to the observation points for both datasets is 1000–4000 mm. The noise levels are the same as those in the shield case.

#### 3.2.2. Result of Fisheye Camera Case

**Figure 11:**
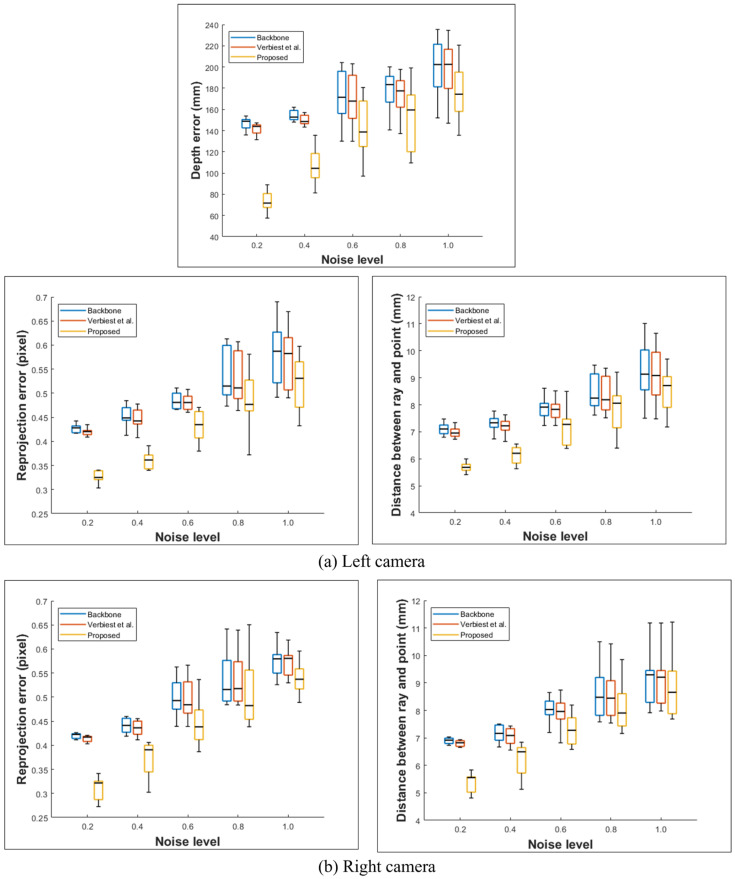
Statistical results (min, max, and standard deviation) for the fisheye case #1 dataset [22].

**Figure 12:**
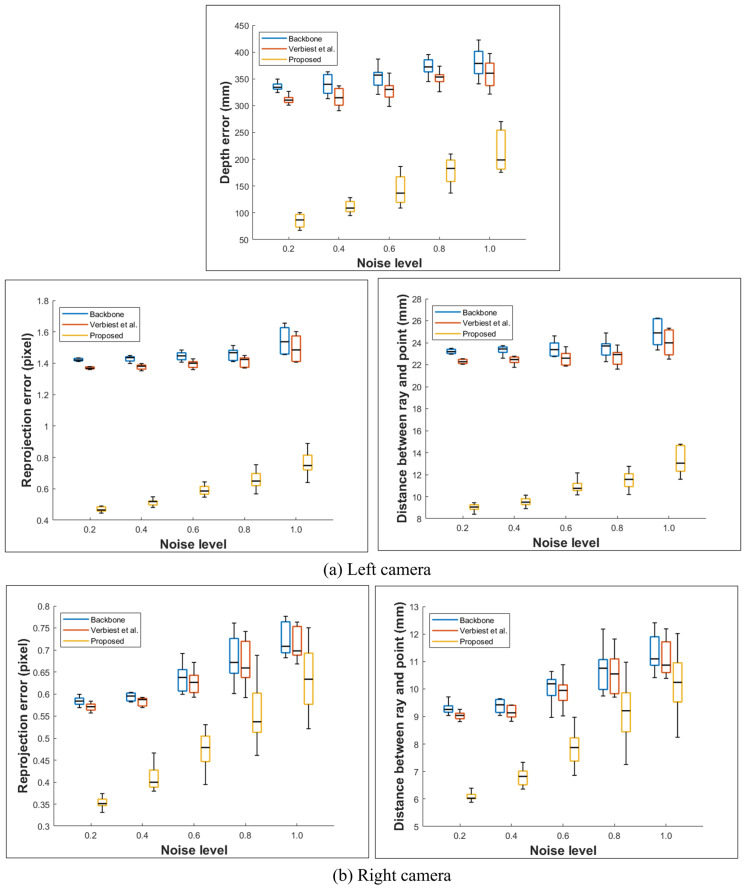
Statistical results (min, max, and standard deviation) for the fisheye case #2 dataset [22].

Table 3 and Table 4 and Figure 11 and Figure 12 show the results of each approach for the fisheye camera. As the noise level increases, the error for the approach of Miraldo et al. [21] increases significantly compared with those of the other methods, and thus we exclude dthis camera model. As in the shield case analysis, the moment and direction in Pl$\ddot{u}$cker coordinates should be perpendicular. However, this assumption does not hold due to the misaligned optical axis or noise. This is likely the reason for the significant increase in the errors.

We compared cases #1 and #2: case #2 has a larger error than that of case #1 because the lens is shifted and tilted, and a larger distortion occurs. As described in the previous configuration, the left and right camera misalignment methods are different, and the error of the left camera is larger. Therefore, tilting the L2 lens has a more significant effect than shifting it. The depth error also increases as the distance between the two rays increases. Similar to the bumpy shield, globally smooth models, such as the backbone and that proposed by Verbiest et al. [22], are inferior to the proposed approach. Moreover, because the method of Verbiest et al. [22] assumes a pinhole camera system, it cannot be applied for a fisheye camera system with a wide field of view.

As in the previous analysis, we performed ten experiments at the same noise level. Figure 11 and Figure 12 show the minimum, maximum, and standard deviation at this noise level. Although the deviation between the proposed method and existing methods decreases as the noise level increases, the error is lower than that in the other methods. Notably, noise levels above 1.0 are unlikely to occur in the natural environment.

### 3.3. Catadioptric Camera with Misaligned Lens

**Figure 13:**
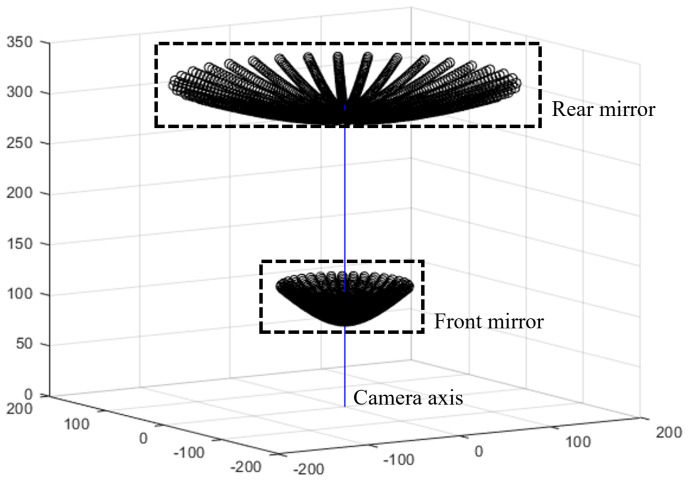
Configuration of catadioptric stereo camera system.

The last camera system involves a catadioptric with two hyperboloid mirrors and one camera observing the reflections of two mirrors, as shown in Figure 13. Although various catadioptric camera configurations are available, we implemented that proposed by Luo et al. [25] owing to its convenience. This configuration has a common perspective camera coupled with two hyperbolic mirrors. The front mirror has a hole in the center, and thus the rear can be seen from this hole.

We evaluated the effects of the tilt and shift, similar to that for the previous fisheye system. The difference with the fisheye camera is that, in this camera system, each mirror has a left and right camera in a pinhole stereo system. Similar to the fisheye camera, in case #1, the mirror was tilted randomly within $1^{\circ }$. In case #2, tilting and shifting were randomly performed within $1^{\circ }$ and 1 mm, respectively. The focal length of the camera is *f* = 1000 (in pixels); the principal point is $c_{x}$ = 500, $c_{y}$ = 500 (in pixels); and the radial and tangential distortions are zero. The image has a width and height of 1000 and 1000 (in pixels).

#### 3.3.1. Calibration Data

The catadioptric system captures images by the reflection of a spherical mirror. Therefore, we simulated the observation points to have a conical shape. These points lie within 1000–5000 mm and the distance was determined experimentally. The training and test points were extracted at 500 and 200 mm intervals, respectively. In addition, we evaluated the performance at the same noise levels as those in the previous camera systems. This camera system is less reliable than the pinhole and fisheye cameras because it is more complex. Therefore, we increased the number of training points over those in the previous two camera systems. A noise level with a random normal distribution of μ=0 and from σ=0 to σ=0.5 was added to the image points, and noise with a random normal distribution of μ=0 and from σ=0 to σ=5 was added to the 3D laser points.

#### 3.3.2. Results for the Catadioptric Camera Case

The results of the reprojection error, distance, and depth error are presented in Table 5 and Table 6. In Figure 13, the front and rear mirrors are the left and right cameras, respectively. The front mirror is more curved than the rear mirror given the field of view of the image sensor. Therefore, the left camera has a larger error than the right camera.

Moreover, because this camera system captures images reflected in mirrors, convergence is not achieved when optimizing the parameters of the backbone, unlike the other systems. Therefore, the noise level in the experiments is smaller than in the different environments.

As noise increases, the relative error rate decreases. As mentioned in the introduction, the proposed method is designed to be suitable for near-central camera systems. These results are thought to appear the closer the noise becomes to the non-central camera. Although the relative error rate is lowered, the proposed method is superior to comparison methods and has the advantage of a small amount of computation.

### 3.4. Performance Analysis of the Proposed Camera Model

We show the computational efficiency of the proposed camera model by evaluating the sum of the computation time for forward and backward projection of the existing iterative methods and the proposed method. The dataset used for time measurement had a noise level of μ=0, σ=0.4 for the image points and μ=0, σ=4 for the 3D laser points. The measurement environment was AMD Ryzen 9 5900X 12-Core Processor 3.70 GHz, 128.0 GB RAM, 64-bit Windows 10, and MATLAB R2022b version. Time was measured as the total test time/number of points. We calculated this measured time as a scalar boost factor for a relative comparison with other methods.

The results are summarized in Table 7. We measured the computation time of each method and determined the speed improvement of the proposed method compared to the existing method by calculating the ratio of the computation time of the existing method to that of the proposed method. The proposed method shows significant computational advantages over other methods.

Because the proposed method interpolates the 3D residual vectors calculated in training, it requires less computation than other methods that are iterative in nature. Compared with the pinhole camera system, the other two systems have a wider angle of view, which makes it challenging to correct the ray. In addition, in an environment in which misalignment and noise occur, the optimization inevitably requires more time. Therefore, the method of Verbiest et al. [22], which requires four spline functions for one point, takes the most time, regardless of the number of points.

## 4. Conclusions

We proposed an efficient camera model and its calibration method that can be applied to a near-central camera system where the rays are converged to nearly one point. In this case, the existing central camera model cannot handle the non-central property of the rays. The non-central camera model can cover the non-central camera rays, but results in slow forward or backward projection and is vulnerable to observation noise. To lessen the computational burden and increase the robustness of the noise, we used the residual concept where the camera model is composed of a backbone model (any central camera model) and a residual model that compensates for the error of the backbone model.

To effectively capture the error of the backbone model, we used 3D observations for the calibration data. With the increased availability of 3D measurement devices, such as 3D laser scanners, we can obtain 3D points for calibration at affordable costs. From the 3D points that we observed, we calculated the residual of the backbone model.

The error of the backbone ray can be estimated by using two points: the training 3D laser point and its nearest point on the backbone ray. The vector starting from the nearest point to the 3D laser point is a residual vector. The vector and its starting point were calculated for all of the training 3D points, forming a sparse 3D vector field. The vector field is smoothed and can be converted to a dense 3D vector field using interpolation. We used this dense 3D vector field to compensate for the error of the forward and backward projection of the backbone model and we called it a residual model.

For the forward projection, the 3D point was first modified by the residual model using the dense 3D vector field. Then, the backbone model performed forward projection from the modified 3D point. For the backward projection, the 2D image point was first transformed into the ray using the backbone model. Then, the near and far points on the backbone ray were modified using the residual model. The ray through two points is then the corrected ray.

The experiments were conducted on a camera system that has a transparent shield in front of the camera, a fisheye camera with misaligned lenses, and a catadioptric camera where the lens or mirrors are tilted or moved slightly. Especially for the transparent shield, we made a case of a bumpy shield, where the outer surface of the shield is deformed, resulting in a non-uniform thickness of the shield. These camera systems result in a non-centrality of the rays that cannot be applied to the central camera model.

The proposed camera model shows a promising performance in forward and backward projection, even with the large noise of the observation. Also in the stereo camera settings, the proposed camera model performs well in estimating 3D points. With an increased accuracy, the computation time is largely reduced because of the non-iterative process in forward or backward projection.

Because the proposed camera model uses an interpolation of the 3D vector field, there is an operating range of the camera model. The range of 3D points is the range and the performance depends on the density of the observations in the 3D. In the future, we will try to research a near-central camera model that can operate on the outside of the training 3D points. This requires additional modeling for the extrapolation of the 3D vector field or some constraint about the straightness of the ray.

## Figures and Tables

**Table 1:** Results for transparent shields with different shapes.

Planar transparent shield: training 488 points/test 3948 points.						
Noise level	Backbone	Miraldo et al. [21]	Verbiest et al. [22]	Proposed	Relative error rate	
0	0.03 ${}^{1}$/0.03 ${}^{2}$/3.90 ${}^{3}$	0.02 ${}^{1}$/0.02 ${}^{2}$/0.90 ${}^{3}$	0.03 ${}^{1}$/0.03 ${}^{2}$/3.60 ${}^{3}$	0.02 ${}^{1}$/0.02 ${}^{2}$/1.60 ${}^{3}$	0/0/−77.7	33.3/33.3/55.5
0.2	0.14 ${}^{1}$/0.14 ${}^{2}$/9.80 ${}^{3}$	0.31 ${}^{1}$/0.35 ${}^{2}$/69.6 ${}^{3}$	0.17 ${}^{1}$/0.16 ${}^{2}$/14.0 ${}^{3}$	0.11 ${}^{1}$/0.11 ${}^{2}$/10.5 ${}^{3}$	64.5/68.5/84.9	35.2/31.2/25.0
0.4	0.25 ${}^{1}$/0.25 ${}^{2}$/23.2 ${}^{3}$	1.17 ${}^{1}$/1.45 ${}^{2}$/276.7 ${}^{3}$	0.30 ${}^{1}$/0.30 ${}^{2}$/37.4 ${}^{3}$	0.22 ${}^{1}$/0.22 ${}^{2}$/19.5 ${}^{3}$	81.2/84.8/92.9	26.6/26.6/47.8
0.6	0.40 ${}^{1}$/0.38 ${}^{2}$/36.8 ${}^{3}$	2.73 ${}^{1}$/3.14 ${}^{2}$/581.5 ${}^{3}$	0.49 ${}^{1}$/0.44 ${}^{2}$/62.1 ${}^{3}$	0.35 ${}^{1}$/0.34 ${}^{2}$/31.2 ${}^{3}$	87.1/89.1/94.6	28.5/22.7/49.7
0.8	0.56 ${}^{1}$/0.55 ${}^{2}$/40.2 ${}^{3}$	5.56 ${}^{1}$/5.47 ${}^{2}$/1085.5 ${}^{3}$	0.71 ${}^{1}$/0.67 ${}^{2}$/89.6 ${}^{3}$	0.45 ${}^{1}$/0.48 ${}^{2}$/37.7 ${}^{3}$	91.9/91.2/96.5	36.6/28.3/57.9
1.0	0.69 ${}^{1}$/0.69 ${}^{2}$/63.4 ${}^{3}$	7.93 ${}^{1}$/9.33 ${}^{2}$/1563.4 ${}^{3}$	0.79 ${}^{1}$/0.77 ${}^{2}$/114.3 ${}^{3}$	0.58 ${}^{1}$/0.54 ${}^{2}$/53.5 ${}^{3}$	92.6/94.2/96.5	26.5/29.8/53.1
Spherical transparent shield: training 486 points/test 3913 points						
Noise level	Backbone	Miraldo et al. [21]	Verbiest et al. [22]	Proposed	Relative error rate	
0	0.05 ${}^{1}$/0.05 ${}^{2}$/4.20 ${}^{3}$	0.04 ${}^{1}$/0.03 ${}^{2}$/6.30 ${}^{3}$	0.04 ${}^{1}$/0.04 ${}^{2}$/3.90 ${}^{3}$	0.04 ${}^{1}$/0.04 ${}^{2}$/2.4 ${}^{3}$	0/−33.3/61.9	0/0/38.4
0.2	0.14 ${}^{1}$/0.14 ${}^{2}$/12.2 ${}^{3}$	0.36 ${}^{1}$/0.32 ${}^{2}$/74.0 ${}^{3}$	0.16 ${}^{1}$/0.16 ${}^{2}$/18.3 ${}^{3}$	0.12 ${}^{1}$/0.12 ${}^{2}$/11.9 ${}^{3}$	66.6/62.5/83.9	25.0/25.0/34.9
0.4	0.26 ${}^{1}$/0.25 ${}^{2}$/24.5 ${}^{3}$	1.31 ${}^{1}$/1.47 ${}^{2}$/316.1 ${}^{3}$	0.32 ${}^{1}$/0.31 ${}^{2}$/38.7 ${}^{3}$	0.22 ${}^{1}$/0.21 ${}^{2}$/25.8 ${}^{3}$	83.2/85.7/91.8	31.2/32.2/33.3
0.6	0.45 ${}^{1}$/0.45 ${}^{2}$/32.9 ${}^{3}$	3.28 ${}^{1}$/3.48 ${}^{2}$/710.3 ${}^{3}$	0.57 ${}^{1}$/0.56 ${}^{2}$/62.6 ${}^{3}$	0.35 ${}^{1}$/0.35 ${}^{2}$/32.2 ${}^{3}$	89.3/89.9/95.4	38.6/37.5/95.4
0.8	0.58 ${}^{1}$/0.59 ${}^{2}$/51.4 ${}^{3}$	5.89 ${}^{1}$/6.08 ${}^{2}$/1229.6 ${}^{3}$	0.71 ${}^{1}$/0.68 ${}^{2}$/76.2 ${}^{3}$	0.46 ${}^{1}$/0.47 ${}^{2}$/50.3 ${}^{3}$	92.1/92.2/95.9	35.2/30.8/33.9
1.0	0.79 ${}^{1}$/0.75 ${}^{2}$/70.2 ${}^{3}$	9.55 ${}^{1}$/8.92 ${}^{2}$/1670.1 ${}^{3}$	0.96 ${}^{1}$/0.89 ${}^{2}$/108.6 ${}^{3}$	0.63 ${}^{1}$/0.61 ${}^{2}$/61.2 ${}^{3}$	93.4/93.1/96.3	34.3/31.4/43.6
Bumpy transparent shield: training 518 points/test 3976 points						
Noise level	Backbone	Miraldo et al. [21]	Verbiest et al. [22]	Proposed	Relative error rate	
0	1.44 ${}^{1}$/1.75 ${}^{2}$/317.6 ${}^{3}$	0.64 ${}^{1}$/0.70 ${}^{2}$/111.7 ${}^{3}$	1.27 ${}^{1}$/1.61 ${}^{2}$/316.8 ${}^{3}$	0.59 ${}^{1}$/0.71 ${}^{2}$/78.7 ${}^{3}$	7.81 ${}^{1,4}$/15.7 ${}^{2,4}$/29.5 ${}^{3,4}$	53.5 ${}^{1,5}$/63.3 ${}^{2,5}$/75.1 ${}^{3,5}$
0.2	1.45 ${}^{1}$/1.76 ${}^{2}$/317.4 ${}^{3}$	0.83 ${}^{1}$/0.79 ${}^{2}$/146.0 ${}^{3}$	1.28 ${}^{1}$/1.61 ${}^{2}$/313.0 ${}^{3}$	0.73 ${}^{1}$/0.83 ${}^{2}$/89.9 ${}^{3}$	12.0/−5.0 ${}^{2,4}$/38.4	42.9 ${}^{1,5}$/48.4/71.2 ${}^{3,5}$
0.4	1.49 ${}^{1}$/1.80 ${}^{2}$/323.4 ${}^{3}$	1.69 ${}^{1}$/1.49 ${}^{2}$/280.4 ${}^{3}$	1.34 ${}^{1}$/1.68 ${}^{2}$/319.9 ${}^{3}$	0.87 ${}^{1}$/0.98 ${}^{2}$/104.8 ${}^{3}$	74.0/67.5 ${}^{2,4}$/62.6	35.0/41.6 ${}^{2,5}$/67.2 ${}^{3,5}$
0.6	1.53 ${}^{1}$/1.81 ${}^{2}$/326.5 ${}^{3}$	3.66 ${}^{1}$/3.30 ${}^{2}$/582.1 ${}^{3}$	1.39 ${}^{1}$/1.69 ${}^{2}$/320.8 ${}^{3}$	0.95 ${}^{1}$/1.07 ${}^{2}$/131.7 ${}^{3}$	74.0/67.5/77.3	31.6/36.6/58.9
0.8	1.63 ${}^{1}$/1.90 ${}^{2}$/330.6 ${}^{3}$	5.77 ${}^{1}$/5.73 ${}^{2}$/939.8 ${}^{3}$	1.51 ${}^{1}$/1.78 ${}^{2}$/326.6 ${}^{3}$	1.08 ${}^{1}$/1.19 ${}^{2}$/146.6 ${}^{3}$	81.2/79.2 ${}^{2,4}$/84.4 ${}^{3,4}$	28.4 ${}^{1,5}$/33.1 ${}^{2,5}$/55.1 ${}^{3,5}$
1.0	1.63 ${}^{1}$/1.93 ${}^{2}$/334.1 ${}^{3}$	9.34 ${}^{1}$/9.21 ${}^{2}$/1380.5 ${}^{3}$	1.51 ${}^{1}$/1.84 ${}^{2}$/323.3 ${}^{3}$	1.20 ${}^{1}$/1.29 ${}^{2}$/166.1 ${}^{3}$	87.1 ${}^{1,4}$/85.9 ${}^{2,4}$/87.9 ${}^{3,4}$	20.5 ${}^{1,5}$/29.8 ${}^{2,5}$/48.6 ${}^{3,5}$

${}^{1}$ Left camera reprojection error (unit: pixel); ${}^{2}$ Right camera reprojection error (unit: pixel); ${}^{3}$ Depth error (unit: mm); ${}^{4}$ Reduction error compared to Miraldo et al. [21] (unit: %); ${}^{5}$ Reduction error compared to Verbiest et al. [22] (unit: %).

**Table 2:** Average angle deviation of the moment and direction vectors from $90^{\circ }$ (unit: degrees).

Noise Level	Left Camera	Right Camera
0	0.000234	0.000131
0.4	2.866433	1.710149

**Table 3:** Results for the misaligned #1 case of the fisheye camera.

Reprojection and depth error: training 147 points/test 1444 points				
Noise level	Backbone	Verbiest et al. [22]	Proposed	Relative error rate
0	0.41 ${}^{1}$/0.40 ${}^{2}$/143.5 ${}^{3}$	0.40 ${}^{1}$/0.40 ${}^{2}$/138.5 ${}^{3}$	0.30 ${}^{1}$/0.30 ${}^{2}$/60.4 ${}^{3}$	25.0 ${}^{1,6}$/25.0 ${}^{2,6}$/56.3 ${}^{3,6}$
0.2	0.43 ${}^{1}$/0.42 ${}^{2}$/147.8 ${}^{3}$	0.42 ${}^{1}$/0.41 ${}^{2}$/142.8 ${}^{3}$	0.33 ${}^{1}$/0.31 ${}^{2}$/73.7 ${}^{3}$	21.4 ${}^{1,6}$/24.3 ${}^{2,6}$/48.3 ${}^{3,6}$
0.4	0.45 ${}^{1}$/0.44 ${}^{2}$/152.8 ${}^{3}$	0.44 ${}^{1}$/0.43 ${}^{2}$/148.5 ${}^{3}$	0.36 ${}^{1}$/0.38 ${}^{2}$/106.1 ${}^{3}$	18.1 ${}^{1,6}$/11.6 ${}^{2,6}$/28.5 ${}^{3,6}$
0.6	0.49 ${}^{1}$/0.50 ${}^{2}$/172.9 ${}^{3}$	0.48 ${}^{1}$/0.49 ${}^{2}$/170.7 ${}^{3}$	0.43 ${}^{1}$/0.45 ${}^{2}$/141.3 ${}^{3}$	10.4 ${}^{1,6}$/8.1 ${}^{2,6}$/17.2 ${}^{3,6}$
0.8	0.53 ${}^{1}$/0.55 ${}^{2}$/181.0 ${}^{3}$	0.53 ${}^{1}$/0.55 ${}^{2}$/177.0 ${}^{3}$	0.48 ${}^{1}$/0.51 ${}^{2}$/152.2 ${}^{3}$	9.4 ${}^{1,6}$/7.2 ${}^{2,6}$/14.0 ${}^{3,6}$
1.0	0.58 ${}^{1}$/0.59 ${}^{2}$/198.4 ${}^{3}$	0.57 ${}^{1}$/0.59 ${}^{2}$/195.6 ${}^{3}$	0.52 ${}^{1}$/0.56 ${}^{2}$/176.4 ${}^{3}$	8.7 ${}^{1,6}$/5.0 ${}^{2,6}$/9.8 ${}^{3,6}$
Distance between the estimated ray and 3D point				
Noise level	Backbone	Verbiest et al. [22]	Proposed	Relative error rate
0	6.91 ${}^{4}$/6.72 ${}^{5}$	6.78 ${}^{4}$/6.61 ${}^{5}$	5.37 ${}^{4}$/5.35 ${}^{5}$	20.8 ${}^{1,6}$/19.0 ${}^{2,6}$
0.2	7.10 ${}^{4}$/6.90 ${}^{5}$	6.61 ${}^{4}$/6.79 ${}^{5}$	5.65 ${}^{4}$/5.37 ${}^{5}$	18.9 ${}^{1,6}$/20.9 ${}^{2,6}$
0.4	7.33 ${}^{4}$/7.15 ${}^{5}$	7.22 ${}^{4}$/7.06 ${}^{5}$	6.14 ${}^{4}$/6.29 ${}^{5}$	14.9 ${}^{1,6}$/10.9 ${}^{2,6}$
0.6	7.83 ${}^{4}$/7.96 ${}^{5}$	7.79 ${}^{4}$/7.90 ${}^{5}$	7.24 ${}^{4}$/7.32 ${}^{5}$	7.0 ${}^{1,6}$/10.9 ${}^{2,6}$
0.8	8.46 ${}^{4}$/8.65 ${}^{5}$	8.35 ${}^{4}$/8.61 ${}^{5}$	7.79 ${}^{4}$/8.15 ${}^{5}$	6.7 ${}^{1,6}$/5.3 ${}^{2,6}$
1.0	9.19 ${}^{4}$/9.24 ${}^{5}$	9.08 ${}^{4}$/9.22 ${}^{5}$	8.58 ${}^{4}$/8.85 ${}^{5}$	5.5 ${}^{1,6}$/4.0 ${}^{2,6}$

${}^{1}$ Left camera reprojection error (unit: pixel); ${}^{2}$ Right camera reprojection error (unit: pixel); ${}^{3}$ Depth error (unit: mm); ${}^{4}$ Left camera distance (unit: mm); ${}^{5}$ Right camera distance (unit: mm); ${}^{6}$ Reduction error compared to Verbiest et al. [22] (unit: %).

**Table 4:** Results for the misaligned #2 case of the fisheye camera.

Reprojection and depth error: training 147 points/test 1444 points				
Noise level	Backbone	Verbiest et al. [22]	Proposed	Relative error rate
0	1.41 ${}^{1}$/0.57 ${}^{2}$/334.2 ${}^{3}$	1.36 ${}^{1}$/0.56 ${}^{2}$/309.6 ${}^{3}$	0.42 ${}^{1}$/0.29 ${}^{2}$/73.5 ${}^{3}$	58.8 ${}^{1,6}$/48.2 ${}^{2,6}$/76.2 ${}^{3,6}$
0.2	1.42 ${}^{1}$/0.58 ${}^{2}$/335.2 ${}^{3}$	1.37 ${}^{1}$/0.57 ${}^{2}$/311.0 ${}^{3}$	0.47 ${}^{1}$/0.35 ${}^{2}$/85.7 ${}^{3}$	65.6 ${}^{1,6}$/38.6 ${}^{2,6}$/72.4 ${}^{3,6}$
0.4	1.43 ${}^{1}$/0.61 ${}^{2}$/341.0 ${}^{3}$	1.38 ${}^{1}$/0.59 ${}^{2}$/314.6 ${}^{3}$	0.52 ${}^{1}$/0.41 ${}^{2}$/110.9 ${}^{3}$	62.3 ${}^{1,6}$/30.5 ${}^{2,6}$/64.7 ${}^{3,6}$
0.6	1.45 ${}^{1}$/0.68 ${}^{2}$/371.7 ${}^{3}$	1.41 ${}^{1}$/0.67 ${}^{2}$/350.9 ${}^{3}$	0.59 ${}^{1}$/0.47 ${}^{2}$/142.5 ${}^{3}$	57.5 ${}^{1,6}$/25.4 ${}^{2,6}$/57.0 ${}^{3,6}$
0.8	1.46 ${}^{1}$/0.68 ${}^{2}$/371.7 ${}^{3}$	1.41 ${}^{1}$/0.67 ${}^{2}$/350.9 ${}^{3}$	0.66 ${}^{1}$/0.56 ${}^{2}$/178.5 ${}^{3}$	53.1 ${}^{1,6}$/16.4 ${}^{2,6}$/49.1 ${}^{3,6}$
1.0	1.58 ${}^{1}$/0.74 ${}^{2}$/397.8 ${}^{3}$	1.52 ${}^{1}$/0.72 ${}^{2}$/371.1 ${}^{3}$	0.80 ${}^{1}$/0.64 ${}^{2}$/225.5 ${}^{3}$	47.3 ${}^{1,6}$/11.1 ${}^{2,6}$/39.2 ${}^{3,6}$
Distance between the ray and 3D point				
Noise level	Backbone	Verbiest et al. [22]	Proposed	Relative error rate
0	23.10 ${}^{4}$/9.19 ${}^{5}$	22.23 ${}^{4}$/8.94 ${}^{5}$	7.85 ${}^{4}$/5.20 ${}^{5}$	64.6 ${}^{4,6}$/41.8 ${}^{5,6}$
0.2	23.22 ${}^{4}$/9.30 ${}^{5}$	22.30 ${}^{4}$/9.06 ${}^{5}$	9.01 ${}^{4}$/6.11 ${}^{5}$	59.6/32.5 ${}^{5,6}$
0.4	23.33 ${}^{4}$/9.58 ${}^{5}$	22.41 ${}^{4}$/9.16 ${}^{5}$	9.51 ${}^{4}$/6.79 ${}^{5}$	57.5 ${}^{4,6}$/25.8 ${}^{5,6}$
0.6	23.48 ${}^{4}$/10.08 ${}^{5}$	22.58 ${}^{4}$/9.88 ${}^{5}$	10.80 ${}^{4}$/7.94 ${}^{5}$	52.1 ${}^{4,6}$/19.6 ${}^{5,6}$
0.8	23.48 ${}^{4}$/10.71 ${}^{5}$	23.67 ${}^{4}$/10.57 ${}^{5}$	11.47 ${}^{4}$/9.08 ${}^{5}$	49.4 ${}^{4,6}$/14.1 ${}^{5,6}$
1.0	25.56 ${}^{4}$/11.54 ${}^{5}$	24.52 ${}^{4}$/11.32 ${}^{5}$	13.79 ${}^{4}$/10.11 ${}^{5}$	43.7 ${}^{4,6}$/10.6 ${}^{5,6}$

${}^{1}$ Left camera reprojection error (unit: pixel); ${}^{2}$ Right camera reprojection error (unit: pixel); ${}^{3}$ Depth error (unit: mm); ${}^{4}$ Left camera distance (unit: mm); ${}^{5}$ Right camera distance (unit: mm); ${}^{6}$ Reduction error compared to Verbiest et al. [22] (unit: %).

**Table 5:** Results for the misaligned #1 case of the catadioptric camera.

Reprojection and depth error: training 827 points/test 2970 points				
Noise level	Backbone	Verbiest et al. [22]	Proposed	Relative error rate
0	0.01 ${}^{1}$/0.06 ${}^{2}$/18.1 ${}^{3}$	0.01 ${}^{1}$/0.05 ${}^{2}$/16.3 ${}^{3}$	0.01 ${}^{1}$/0.02 ${}^{2}$/7.4 ${}^{3}$	0 ${}^{1,6}$/60 ${}^{2,6}$/54.6 ${}^{3,6}$
0.1	0.03 ${}^{1}$/0.06 ${}^{2}$/25.3 ${}^{3}$	0.03 ${}^{1}$/0.06 ${}^{2}$/24.7 ${}^{3}$	0.03 ${}^{1}$/0.04 ${}^{2}$/24.3 ${}^{3}$	0 ${}^{1,6}$/33.3 ${}^{2,6}$/1.6 ${}^{3,6}$
0.2	0.04 ${}^{1}$/0.07 ${}^{2}$/25.8 ${}^{3}$	0.04 ${}^{1}$/0.07 ${}^{2}$/25.4 ${}^{3}$	0.04 ${}^{1}$/0.06 ${}^{2}$/24.9 ${}^{3}$	0 ${}^{1,6}$/14.2 ${}^{2,6}$/1.9 ${}^{3,6}$
0.3	0.05 ${}^{1}$/0.08 ${}^{2}$/36.2 ${}^{3}$	0.05 ${}^{1}$/0.08 ${}^{2}$/35.8 ${}^{3}$	0.05 ${}^{1}$/0.07 ${}^{2}$/35.6 ${}^{3}$	0 ${}^{1,6}$/12.5 ${}^{2,6}$/0.5 ${}^{3,6}$
0.4	0.06 ${}^{1}$/0.10 ${}^{2}$/37.6 ${}^{3}$	0.06 ${}^{1}$/0.10 ${}^{2}$/35.7 ${}^{3}$	0.05 ${}^{1}$/0.09 ${}^{2}$/34.5 ${}^{3}$	16.6 ${}^{1,6}$/10.0 ${}^{2,6}$/0.5 ${}^{3,6}$
0.5	0.08 ${}^{1}$/0.12 ${}^{2}$/54.4 ${}^{3}$	0.08 ${}^{1}$/0.11 ${}^{2}$/53.1 ${}^{3}$	0.08 ${}^{1}$/0.11 ${}^{2}$/52.2 ${}^{3}$	0 ${}^{1,6}$/0 ${}^{2,6}$/1.6 ${}^{3,6}$
Distance between the estimated ray and 3D point				
Noise level	Backbone	Verbiest et al. [22]	Proposed	Relative error rate
0	0.64 ${}^{4}$/0.72 ${}^{5}$	0.64 ${}^{4}$/0.72 ${}^{5}$	0.35 ${}^{4}$/0.30 ${}^{5}$	45.3 ${}^{4,6}$/58.3 ${}^{5,6}$
0.1	0.84 ${}^{4}$/0.82 ${}^{5}$	0.83 ${}^{4}$/0.78 ${}^{5}$	0.77 ${}^{4}$/0.58 ${}^{5}$	7.2 ${}^{4,6}$/25.6 ${}^{5,6}$
0.2	1.05 ${}^{4}$/0.91 ${}^{5}$	1.05 ${}^{4}$/0.89 ${}^{5}$	1.02 ${}^{4}$/0.77 ${}^{5}$	2.8 ${}^{4,6}$/13.4 ${}^{5,6}$
0.3	1.51 ${}^{4}$/1.06 ${}^{5}$	1.51 ${}^{4}$/1.05 ${}^{5}$	1.45 ${}^{4}$/0.98 ${}^{5}$	3.9 ${}^{4,6}$/6.6 ${}^{5,6}$
0.4	1.56 ${}^{4}$/1.33 ${}^{5}$	1.55 ${}^{4}$/1.24 ${}^{5}$	1.47 ${}^{4}$/1.22 ${}^{5}$	5.1 ${}^{4,6}$/1.6 ${}^{5,6}$
0.5	2.30 ${}^{4}$/1.60 ${}^{5}$	2.30 ${}^{4}$/1.47 ${}^{5}$	2.22 ${}^{4}$/1.34 ${}^{5}$	3.4 ${}^{4,6}$/8.8 ${}^{5,6}$

${}^{1}$ Left camera reprojection error (unit: pixel); ${}^{2}$ Right camera reprojection error (unit: pixel); ${}^{3}$ Depth error (unit: mm); ${}^{4}$ Left camera distance (unit: mm); ${}^{5}$ Right camera distance (unit: mm); ${}^{6}$ Reduction error compared to Verbiest et al. [22] (unit: %).

**Table 6:** Results for the misaligned #2 case of the catadioptric camera.

Reprojection and depth error: training 826 points/test 2965 points				
Noise level	Backbone	Verbiest et al. [22]	Proposed	Relative error rate
0	0.08 ${}^{1}$/0.09 ${}^{2}$/54.0 ${}^{3}$	0.08 ${}^{1}$/0.07 ${}^{2}$/50.3 ${}^{3}$	0.02 ${}^{1}$/0.02 ${}^{2}$/22.1 ${}^{3}$	75.0 ${}^{1,6}$/71.4 ${}^{2,6}$/56.0 ${}^{3,6}$
0.1	0.09 ${}^{1}$/0.10 ${}^{2}$/59.7 ${}^{3}$	0.09 ${}^{1}$/0.08 ${}^{2}$/58.0 ${}^{3}$	0.05 ${}^{1}$/0.06 ${}^{2}$/46.3 ${}^{3}$	44.4 ${}^{1,6}$/25.0 ${}^{2,6}$/20.1 ${}^{3,6}$
0.2	0.10 ${}^{1}$/0.13 ${}^{2}$/68.2 ${}^{3}$	0.10 ${}^{1}$/0.11 ${}^{2}$/64.9 ${}^{3}$	0.07 ${}^{1}$/0.09 ${}^{2}$/49.3 ${}^{3}$	30.0 ${}^{1,6}$/18.1 ${}^{2,6}$/24.0 ${}^{3,6}$
0.3	0.10 ${}^{1}$/0.15 ${}^{2}$/73.7 ${}^{3}$	0.10 ${}^{1}$/0.13 ${}^{2}$/70.0 ${}^{3}$	0.08 ${}^{1}$/0.11 ${}^{2}$/59.3 ${}^{3}$	20.0 ${}^{1,6}$/18.1 ${}^{2,6}$/24.0 ${}^{3,6}$
0.4	0.11 ${}^{1}$/0.16 ${}^{2}$/77.0 ${}^{3}$	0.11 ${}^{1}$/0.14 ${}^{2}$/71.6 ${}^{3}$	0.10 ${}^{1}$/0.13 ${}^{2}$/66.9 ${}^{3}$	9.0 ${}^{1,6}$/15.3 ${}^{2,6}$/6.5 ${}^{3,6}$
0.5	0.14 ${}^{1}$/0.18 ${}^{2}$/91.9 ${}^{3}$	0.14 ${}^{1}$/0.15 ${}^{2}$/87.4 ${}^{3}$	0.12 ${}^{1}$/0.14 ${}^{2}$/83.5 ${}^{3}$	14.2 ${}^{1,6}$/6.6 ${}^{2,6}$/4.4 ${}^{3,6}$
Distance between the estimated ray and 3D point				
Noise level	Backbone	Verbiest et al. [22]	Proposed	Relative error rate
0	2.39 ${}^{4}$/1.25 ${}^{5}$	2.37 ${}^{4}$/1.04 ${}^{5}$	0.70 ${}^{4}$/0.28 ${}^{5}$	70.4 ${}^{4,6}$/73.0 ${}^{5,6}$
0.1	2.69 ${}^{4}$/1.35 ${}^{5}$	2.67 ${}^{4}$/1.11 ${}^{5}$	1.47 ${}^{4}$/0.66 ${}^{5}$	44.9 ${}^{4,6}$/40.5 ${}^{5,6}$
0.2	2.90 ${}^{4}$/1.77 ${}^{5}$	2.88 ${}^{4}$/1.48 ${}^{5}$	1.98 ${}^{4}$/1.09 ${}^{5}$	31.2 ${}^{4,6}$/26.3 ${}^{5,6}$
0.3	2.92 ${}^{4}$/2.07 ${}^{5}$	2.91 ${}^{4}$/1.72 ${}^{5}$	2.32 ${}^{4}$/1.44 ${}^{5}$	20.2 ${}^{4,6}$/16.2 ${}^{5,6}$
0.4	3.08 ${}^{4}$/2.11 ${}^{5}$	3.07 ${}^{4}$/1.84 ${}^{5}$	2.74 ${}^{4}$/1.71 ${}^{5}$	10.7 ${}^{4,6}$/7.0 ${}^{5,6}$
0.5	3.87 ${}^{4}$/2.36 ${}^{5}$	3.85 ${}^{4}$/1.93 ${}^{5}$	3.52 ${}^{4}$/1.76 ${}^{5}$	8.5 ${}^{4,6}$/8.8 ${}^{5,6}$

${}^{1}$ Left camera reprojection error (unit: pixel); ${}^{2}$ Right camera reprojection error (unit: pixel); ${}^{3}$ Depth error (unit: mm); ${}^{4}$ Left camera distance (unit: mm); ${}^{5}$ Right camera distance (unit: mm); ${}^{6}$ Reduction error compared to Verbiest et al. [22] (unit: %).

**Table 7:** Relative performance analysis of the proposed method with respect to the existing method. For example, in the first row and third column, the proposed camera model is 251.5 times faster than Miraldo el al. [21] in a fisheye camera system (unit: scale factor). The computation time is calculated by sum of the forward and backward projection.

Camera System	Number of Points	w.r.t. Miraldo et al. [21]	w.r.t. Verbiest et al. [22]
Fisheye	1444	251.5	953.5
Catadioptric	2970	88.1	192.1
Pinhole ${}^{1}$	3913	161.8	236.8
Pinhole ${}^{2}$	3948	151.2	247.0
Pinhole ${}^{3}$	3967	195.0	296.0

${}^{1}$ Spherical shield; ${}^{2}$ Planar shield; ${}^{3}$ Bumpy shield.

## Data Availability

Not applicable.
